# Characteristics of small pancreatic neuroendocrine tumors and risk factors for invasion and metastasis

**DOI:** 10.3389/fendo.2023.1140873

**Published:** 2023-03-20

**Authors:** Wentong Mei, Feng Cao, Jiongdi Lu, Chang Qu, Zhen Fang, Jia Li, Fei Li

**Affiliations:** ^1^ Department of General Surgery, Xuanwu Hospital, Capital Medical University, Beijing, China; ^2^ Clinical Center for Acute Pancreatitis, Capital Medical University, Beijing, China

**Keywords:** pancreatic neuroendocrine tumors, pNETs, tumor biological characteristics, surgery, risk factor

## Abstract

**Background:**

The number of people with small pancreatic neuroendocrine tumors (pNETs) (tumors with a diameter less than or equal to 2 cm) is gradually increasing, but the selection of treatment strategy is still controversial. Our aim was to characterize small pNETs with a poor prognosis and to define the impact of aggressive small pNETs on survival and the risk factors for the development of invasive disease.

**Methods:**

Patients with pNETs diagnosed between 2004 and 2019 and a tumor diameter of 2 cm or less were selected from the SEER Registry. Kaplan–Meier survival analysis was used to identify the factors affecting patient survival, and binary logistic regression was used to identify the associated risk factors.

**Results:**

A total of 3261 patients with pNETs were enrolled in the study. Both older and younger patients benefited from surgery. Regional invasion occurred in 10% of the patients, and distant metastases occurred in 9% of the patients, but in both categories, those who underwent surgery had better survival outcomes than those who did not. There was no difference in survival between patients with a tumor diameter of 1–2 cm and those with a tumor diameter of less than 1 cm, and there was no difference in survival between patients with functional and nonfunctional small pNETs. However, the survival of patients with pNETs in the head of the pancreas was worse than that of patients with tumors in other parts of the pancreas. Survival was worse in elderly patients and in those with poorly differentiated and undifferentiated tumors. Lymphatic metastasis, regional invasion, and distant metastasis all worsened the prognosis of patients. The presence or absence of neuroendocrine function, the degree of tumor differentiation, and the location of the tumor were associated with the risk of lymphatic metastasis and regional invasion; the risk factors for distant metastasis were associated with the degree of differentiation and tumor location.

**Conclusion:**

The pNETs ≤ 2 cm in diameter could be still aggressive, and patient prognosis worsens after invasive disease develops. Attention to the characteristics of aggressive tumors can improve patient survival.

## 1 Introduction

Pancreatic neuroendocrine tumors (pNETs) are a class of heterogeneous tumors that originate from pancreatic neuroendocrine cells and have pathological neuroendocrine features. They are rarer than other pancreatic cancers, accounting for approximately 3–5% of all pancreatic tumors (1). PNETs can be clinically classified as functional or nonfunctional depending on whether they release symptom-producing hormones: most of them are nonfunctioning and asymptomatic (2). About 5% of pNETs induced by hereditary diseases (3), such as type 1 multiple endocrine neoplasia, von Hippel–Lindau disease and tuberous sclerosis, the vast majority of pNETs are sporadic tumors. The incidence of pNETs has gradually increased in recent years, particularly as a result of improvements in diagnostic technology. An increasing number of patients with pNETs ≤2 cm in diameter have been identified, and the proportion of such patients among all pNET patients has gradually increased (4). The pNETs are highly heterogeneous; some of them progress rapidly, and surgery is still the main treatment method with curative intent (5). The traditional view is that the smaller the pNETs are, the less aggressive their biological behavior. Some early studies, upon which the initial ENETS treatment guidelines were based, indicated that the nonsurgical method of observation and follow-up for small pNETs was safe and reliable (6, 7). However, the management of pNETs ≤ 2 cm in diameter is still controversial because some tumors of this type are aggressive. Some studies have reported that the incidence of lymph node metastasis in patients with these small pNETs can reach 24–27% (8, 9), and the proportion of patients with distant metastasis (M1) can be as high as 7.6% (10). Even though some literature reviews or meta-analyses have suggested that small tumors may be less malignant than larger ones, it is difficult to justify the selection of observation as a course of action based on tumor size alone. Since there is still risk involved in the surgical treatment of pNETs, the identification of relatively more aggressive pNETs (≤2 cm) and the related risk factors and the clarification of the benefits of surgery would be helpful with regard to clinical decision-making. In addition, lymph node metastasis is an important risk factor affecting patient prognosis. According to the American Joint Committee on Cancer (AJCC), lymph node metastasis can be used to directly classify neuroendocrine tumors as stage III (11). It has been previously reported that positivity for lymph node metastasis is related to tumor size, pathological differentiation and location, while Lopez-Aguiar AG et al. stated that a tumor diameter larger than 2 cm was an independent risk factor for pNET metastasis (12). However, there have been few independent studies on the risk factors for lymph node metastasis in pNETs smaller than 2 cm in diameter. The risks associated with the local progression and distant metastasis of small pNETs are also unclear. Nevertheless, there remain risks associated with the surgical treatment of PNETs, which means that it is important for clinical decision making to determine the benefits of surgery in patients with pNETs smaller than or equal to 2 cm in diameter and identify the relatively more aggressive tumors and their associated risk factors.

The purpose of this study was to summarize the clinical characteristics of patients with pNETs that are ≤2 cm in diameter and comprehensively describe the characteristics of such tumors in a large sample of patients, including the benefits of surgical treatment, the impact of tumor characteristics on survival, and the risk factors for metastasis to lymph nodes and distant organs. These results can describe the clinical characteristics of pNETs smaller than or equal to 2 cm in diameter and help surgeons find the more aggressive tumors.

## 2 Patients and methods

### 2.1 Data and cohort selection

The data for this retrospective cohort study were obtained from the Surveillance, Epidemiology, and End Results (SEER) registry (13), which is an open and accessible public database supported by the National Cancer Center. The study data were retrieved and collected using SEER Stat 8.4.0.1. We screened the data from all patients with pancreatic neuroendocrine tumors recorded from 2004 to 2019 in the SEER registry. PNETs were defined according to the International Classification of Disease for Oncology 3rd edition (ICD-O-3) site codes (which was updated in 2019), including functional pNETs [pancreatic endocrine tumor, benign (8150/0); pancreatic endocrine tumor, malignant (8150/3); insulinoma, malignant (8151/3); glucagonoma, malignant (8152/3); gastrinoma, malignant (8153/3); mixed pancreatic endocrine and exocrine tumor, malignant (8154/3); vipoma, malignant (8155/3); and somatostatinoma, malignant (8156/3)] and nonfunctional pNETs [carcinoid tumor *in situ* (8240/2); carcinoid tumor NOS (8240/3); enterochromaffin cell carcinoid (8241/3); enterochromaffin-like cell tumor, malignant (8242/3); goblet cell carcinoid *in situ* (8243/2); goblet cell carcinoid (8243/3); mixed adenoneuroendocrine carcinoma (8244/3 ICD-O-3 updated); adenocarcinoid tumor (8245/3); neuroendocrine carcinoma *in situ* (8246/2); neuroendocrine carcinoma NOS (8246/3); and atypical carcinoid tumor (8249/3). The inclusion criteria were the availability of complete demographic and epidemiological information and follow-up information, tumor size ≤2 cm, and the availability of complete surgical and tumor location information.

### 2.2 Statistical analysis

Student’s t test, the Mann–Whitney U test, the chi-square test and Fisher’s exact test were used as appropriate when analyzing the baseline data and tumor biological information. Univariate or multivariate logistic regression analyses were used to assess the risk factors for tumor invasion. The variables analyzed included basic information of the patient (gender, age, ethnicity) and the tumor characteristics (degree of tumor differentiation, size, location, and endocrine function). The Kaplan–Meier method and log-rank test were used for survival analysis and comparisons between groups. Survival months and vital status recode in the SEER registry were used to evaluate the time on the X-axis and the survival probability on the Y-axis of the survival curves, respectively. Overall survival (OS) and cancer-specific survival (CSS) were calculated. When risk factor (e.g., pathological grade, location) data included cases with unrecorded information, those cases were excluded.

The *p* values are presented with the hazard ratios and 95% confidence intervals (CIs), and statistical significance was determined by a p value <0.05. R3.6.4 and R package were used for all statistical analyses.

## 3 Results

### 3.1 The patients in surgical group have better survival

A total of 3261 patients who met the inclusion criteria were identified from all of the data. First, patients were divided into two groups based on whether they had undergone surgery: 2233 were in the surgical group, and 1028 were in the nonsurgical group. The baseline characteristics of the two groups were compared. There were no differences according to sex or race. The mean tumor sizes in patients treated with surgery and those not treated with surgery were similar, and there was no significant difference. In the total patient population, there were more patients with nonfunctional tumors than functional tumors, with the former group accounting for approximately 93% of the patients. Patients with functional tumors accounted for 8% of the surgical group, which is higher than the 4% in the nonsurgical group. Patients in the surgical group were younger than those in the nonsurgical group, and patients older than 65 years accounted for 41% of those in the surgical group, compared with 58% of the nonsurgical group. Tumors located in the tail of the pancreas were the most common in the surgery group (38%), while tumors in the head of the pancreas were the most common in the nonsurgical group (28%). Approximately 80% of all patients had localized tumors, 10% had regional progression, 9% had remote metastasis and 1% lacked data on progression. Tumor development was more limited in the patients in the surgical group, with only 3% of patients assigned to M1, compared with the 22% of patients who had distant metastasis in the nonsurgical group (**Table 1**).

**Table 1:** Demographic and patient characteristics in the cohort.

Parameter	Total (N,%) N=3261	Surgical group N=2233	Nonsurgical group N=1028	P
**Age(years)**				**<0.001**
<65	1757 (54)	1323(59)	434(42)	
≥65	1504(46)	910(41)	594(58)	
**Sex**				**0.841**
Female	1612(49)	1107(49)	505(49)	
Male	1649(51)	1126(51)	523(51)	
**Race**				**0.529**
White	2582(79)	1759(79)	823(80)	
Black	336(10)	230(10)	106(11)	
Others	343(11)	244(11)	99(9)	
**Histology**				**<0.001**
Functional	230(7)	186(8)	44(4)	
Nonfunctional	3031(93)	2047(92)	984(96)	
**Tumor stage**				**<0.001**
Localized	2615(80)	1869(84)	746(73)	
Regional	326(10)	290(12)	36(3)	
Distant	286(9)	63(3)	223(22)	
Unstaged	34(1)	11(1)	23(2)	
**Tumor size**				**0.337**
0-10mm	963(30)	648(29)	315(31)	
11-20mm	2298(70)	1585(71)	713(69)	
Median (IQR) mm	14 (10,17)	14 (10,17)	13 (10,17)	0.346
**Tumor location**				**<0.001**
Pancreatic head	835(26)	537(24)	298(29)	
Pancreatic body	709(22)	484(22)	225(22)	
Pancreatic tail	1144(35)	853(38)	291(28)	
Overlapping lesion	135(4)	87(4)	48(5)	
Other location	438(13)	272(12)	166(16)	
**Grade**				**<0.001**
Well differentiated(G1)	2006(62)	1750(78)	256(25)	
Moderately differentiated(G2)	277(9)	229(10)	48(5)	
Poorly differentiated(G3)	41(1)	22(1)	19(2)	
Undifferentiated(G4)	15(0)	8(0)	7(1)	
Unknown	922(28)	224(11)	698(67)	

IQR, interquartile range. The bold values mean the difference between the groups was statistically significant.

**Figure 1:**
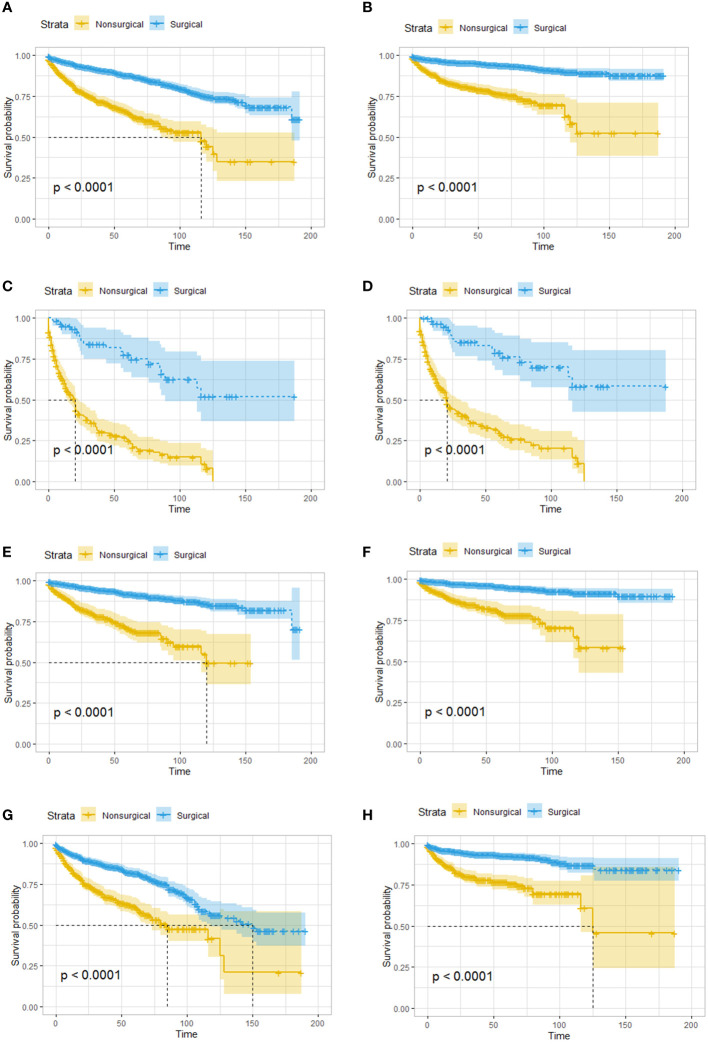
Survival analysis based on surgery: patients in the surgical group had better OS and CSS, regardless of age and tumor metastasis. OS **(A)** and CSS **(B)** of all the patients. OS **(C)** and CSS **(D)** of patients with distant metastatic tumor. OS **(E)** and CSS **(F)** of patients younger than 65 years old. OS **(G)** and CSS **(H)** of patients greater than or equal 65 years old. OS, overall survival; CSS, cancer-specific survival.

Kaplan–Meier survival analysis was performed, and survival was compared between the two treatment groups. Overall, patients in the surgical group had better overall survival (OS) and cancer-specific survival (CSS) than those in the nonsurgical group (**Figures 1A, B**). Even for patients with distant metastasis, surgery still yielded benefits, and the OS and CSS was significantly worse in the nonsurgical group (**Figures 1C, D**). Given that the age difference between the surgical group and the nonsurgical group could affect their survival outcomes, we evaluated elderly and young patients separately, and the results showed that the OS and CSS of patients who received surgery were better than those who received nonsurgical treatment, regardless of whether they were younger than (**Figures 1E, F**) or older than 65 years (**Figures 1G, H**).

### 3.2 Tumor size and endocrine function were not associated with survival, and the patients with pancreatic head tumors had worst survival

**Figure 2:**
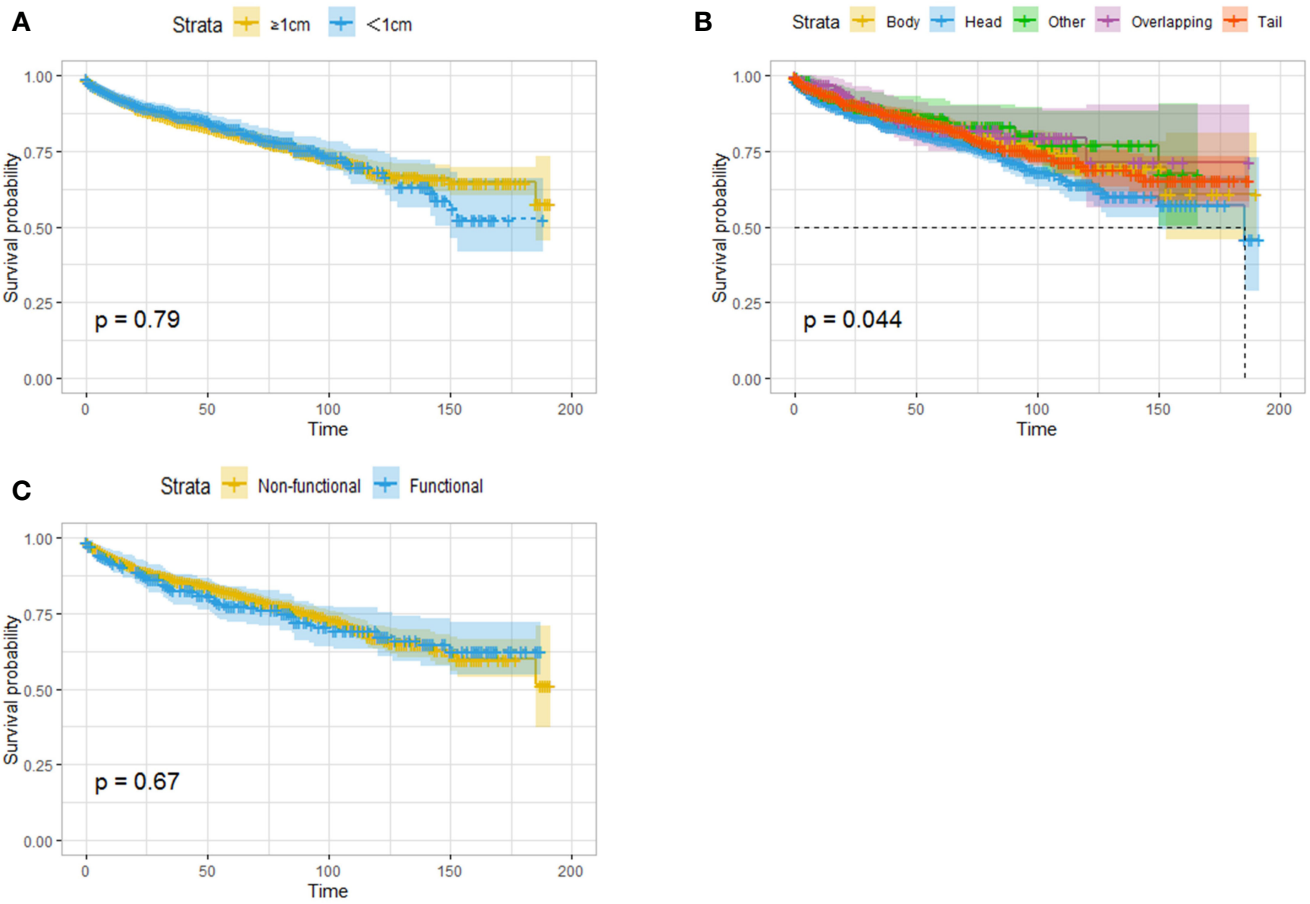
Survival analysis (OS) based on the size of tumor **(A)**, the location of the tumor **(B)** and the functional status **(C)**. Tumor size and endocrine function were not associated with survival, and the patients with pancreatic head tumors had worst survival.

Given that previous studies have reported that a tumor diameter smaller than 1 cm may be associated with better survival, survival analysis was performed on patients stratified by a whether the tumor size was larger than 1 cm, and no difference in OS was found between the two groups (**Figure 2A**). Tumor location was related to prognosis, and pancreatic head tumors were associated with the worst survival (**Figure 2B**). In addition, whether the tumor was functional did not affect OS (**Figure 2C**).

### 3.3 Elderly patients have lower OS and CSS, and the degree of tumor differentiation affects the prognosis

**Figure 3:**
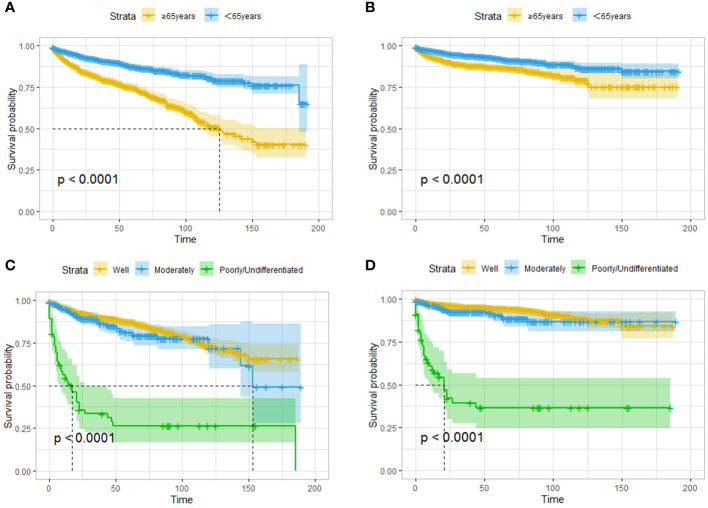
Survival analysis based on age: OS **(A)** and CSS **(B)** of all the patients. Survival analysis based on the degree of tumor differentiation: OS **(C)** and CSS **(D)** of all the patients. Elderly patients have lower OS and CSS, and the degree of tumor differentiation affects the prognosis.

A total of 1504 patients were 65 years or older. In the survival analysis, these elderly patients had worse OS and CSS than the younger patients (**Figures 3A, B**). Tumor specimens from 2339 patients were pathologically examined and graded, and 56 patients (2.4%) had poorly differentiated or undifferentiated tumors; these patients had significantly reduced OS and CSS (**Figures 3C, D**). In addition, 2006 patients had well-differentiated tumors, and 277 patients had moderately differentiated tumors.

### 3.4 Positivity for lymph node metastasis, local tumor invasion and distant metastasis were associated with reduced survival

The results showed that pNETs smaller than or equal to 2 cm in diameter were still invasive. In the surgical group, lymph node excision and pathological findings were recorded in 1612 patients, 216 of whom had one or more positive lymph nodes.

**Figure 4:**
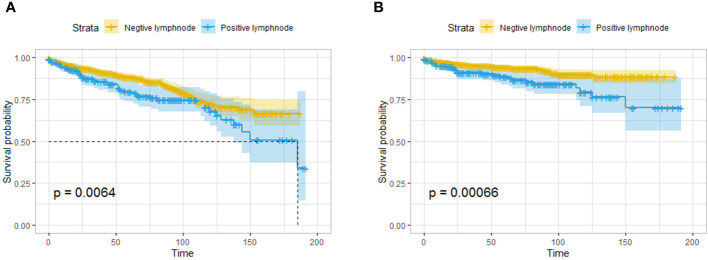
Survival analysis based on the pathological findings of lymph nodes: OS **(A)** and CSS **(B)** of all the patients. The patients with lymph node metastasis had worse OS and CSS than those without lymph node metastasis.

In the Kaplan–Meier survival analysis, patients with lymph node metastasis had significantly worse OS and CSS than those without lymph node metastasis (**Figures 4A, B**). Logistic regression was performed on the patient clinical characteristics to identify the risk factors for lymph node metastasis. The presence of functional tumors, poorly differentiated tumors, and tumors in the head of the pancreas were risk factors for lymph node metastasis in both univariate and multivariate regression analyses. The odds ratio (OR) increased with decreasing degree of tumor differentiation. The risk of lymph node metastasis for tumors in the body and tail of the pancreas is lower than that for tumors in the head of the pancreas (**Table 2**).

**Table 2:** Logistic regression analysis of clinicopathological factors associated with lymph node metastasis.

	Univariable	P	Multivariable	P
	OR (95% CI)		OR (95% CI)	
Age,years				
<65	Ref			
≥65	0.994(0.961-1.028)	0.740		
Sex				
Male	Ref			
Female	1.000(0.967-1.034)	0.992		
Race				
White	Ref			
Black	1.029(0.977-1.084)	0.281		
Others	0.955(0.904-1.009)	0.105		
Grade				
Well differentiated(G1)	Ref		Ref	
Moderately differentiated(G2)	1.092(1.038-1.149)	***<0.001***	1.083(1.028-1.140)	***0.003***
Poorly differentiated(G3)	1.273(1.102-1.471)	***0.001***	1.186(1.026-1.372)	***0.021***
Undifferentiated(G4)	1.589 (1.246-2.026)	***<0.001***	1.527(1.204-1.937)	***<0.001***
Tumor size				
0-10mm	Ref			
11-20mm	1.036(0.998-1.075)	0.062		
Tumor location				
Pancreatic head	Ref		Ref	
Pancreatic body	0.877(0.836-0.921)	***<0.001***	0.884(0.841-0.928)	***<0.001***
Pancreatic tail	0.863(0.828-0.899)	***<0.001***	0.884(0.848-0.921)	***<0.001***
Overlapping lesion	0.955(0.877-1.033)	0288	0.955(0.875-1.042)	0.307
Other location	0.975(0.875-1.062)	0.561	0.956(0.875-1.043)	0.310
Histology				
Nonfunctional	Ref		Ref	
Functional	1.107(1.042-1.176)	***0.001***	1.100(1.025-1.180)	***0.008***

CI, confidence intervals. The bold values mean the difference between the groups was statistically significant.

**Figure 5:**
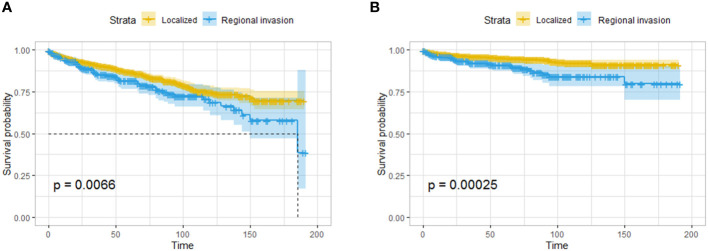
Survival analysis based on the state of regional invasion of the tumor: OS **(A)** and CSS **(B)** of all the patients. The patients with regional invasion had worse OS and CSS than those with localized tumors.

A total of 326 patients with regional invasion had worse OS and CSS than those with localized tumors (**Figures 5A, B**). Logistic regression analysis was performed on the risk of regional invasion in all patients. According to univariate regression, being Black, having a moderately or poorly differentiated tumor, having a tumor in the head of the pancreas, having a tumor larger than 1 cm, and having a tumor with endocrine function were risk factors for local invasion, while having a tumor in the body or tail of the pancreas was associated with a reduced risk of local invasion. According to multivariate regression, the risk factors for local invasion were the same as those identified in univariate regression except for being Black; the protective factors were the same as those identified in univariate regression (**Table 3**).

**Table 3:** Logistic regression analysis of clinicopathological factors associated with regional extension.

	Univariable	P	Multivariable	P
	OR (95% CI)		OR (95% CI)	
Age,years				
<65	Ref			
≥65	0.988(0.966-1.011)	0.315		
Sex				
Male	Ref			
Female	1.003(0.980-1.026)	0.800		
Race				
White	Ref		Ref	
Black	1.053(1.014-1.094)	***0.008***	1.040(0.993-1.088)	0.094
Others	0.971(0.936-1.008)	0.121	0.963(0.921-1.007)	0.099
Grade				
Well differentiated(G1)	Ref		Ref	
Moderately differentiated(G2)	1.090 (1.045-1.137)	***<0.001***	1.087(1.040-1.135)	***<0.001***
Poorly differentiated(G3)	1.485(1.297-1.699)	***<0.001***	1.459(1.272-1.673)	***<0.001***
Undifferentiated(G4)	1.257 (0.972-1.625)	0.081	1.267(0.959-1.674)	0.096
Tumor size				
0-10mm	Ref		Ref	
11-20mm	1.051(1.025-1.077)	***<0.001***	1.026(0.995-1.058)	0.100
Tumor location				
Pancreatic head	Ref		Ref	
Pancreatic body	0.917(0.887-0.947)	***<0.001***	0.918(0.883-0.955)	***<0.001***
Pancreatic tail	0.916(0.889-0.943)	***<0.001***	0.915(0.884-0.947)	***<0.001***
Overlapping lesion	0.987(0.930-1.047)	0.665	0.984(0.918-1.055)	0.654
Other location	0.968(0.917-1.021)	0.229	0.937(0.878-1.001)	0.054
Histology				
Nonfunctional	Ref		Ref	
Functional	1.087(1.039-1.136)	***<0.001***	1.061(1.001-1.125)	***0.045***

CI, confidence intervals. The bold values mean the difference between the groups was statistically significant.

**Figure 6:**
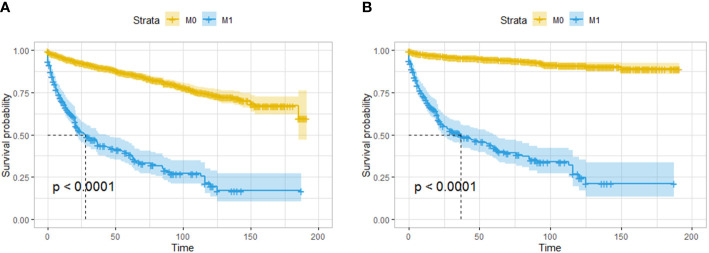
Survival analysis based on the state of distant invasion of the tumor: OS **(A)** and CSS **(B)** of all the patients. The patients with distant metastasis had worse OS and CSS than those without distant metastasis.

A total of 286 patients had distant metastases and were classified as having M1 disease. The OS and CSS of patients with distant metastasis were significantly worse than those with localized or regionally invasive tumors (**Figures 6A, B**). The median OS for patients with M1 tumors was only 28 months. According to univariate logistic regression, female patients, patients with tumors larger than 10 mm in diameter and patients with poorly differentiated tumors had a higher risk of distant metastasis. The occurrence of tumors in the body and tail of the pancreas was associated with a reduced risk of distant metastasis. However, in multivariate logistic regression, an increased risk of distant metastasis was only found to be associated with a reduced degree of pathological differentiation, and only the occurrence of tumors in the body of the pancreas was associated with a lower risk of distant metastasis (**Table 4**).

**Table 4:** Logistic regression analysis of clinicopathological factors associated with distant metastases.

	Univariable	P	Multivariable	P
	OR (95% CI)		OR (95% CI)	
Age,years				
<65	Ref			
≥65	1.002(0.983-1.022)	0.837		
Sex				
Male	Ref		Ref	
Female	1.025(1.006-1.046)	***0.011***	1.015(0.999-1.032)	0.073
Race				
White	Ref			
Black	1.026(0.993-1.060)	0.129		
Others	0.992(0.960-1.025)	0.640		
Grade				
Well differentiated(G1)	Ref		Ref	
Moderately differentiated(G2)	1.055(1.029-1.082)	***<0.001***	1.052(1.025 -1.079)	***<0.001***
Poorly differentiated(G3)	1.542(1.450-1.641)	***<0.001***	1.491(1.400-1.588)	***<0.001***
Undifferentiated(G4)	1.768 (1.597-1.957)	***<0.001***	1.727(1.548-1.928)	***<0.001***
Tumor size				
0-10mm	Ref		Ref	
11-20mm	1.070(1.047-1.093)	***<0.001***	1.014(0.995-1.032)	0.147
Tumor location				
Pancreatic head	Ref		Ref	
Pancreatic body	0.940(0.914-0.967)	***<0.001***	0.976(0.954-0.999)	***0.042***
Pancreatic tail	0.954(0.915-1.037)	***<0.001***	0.988(0.969-1.009)	0.265
verlapping lesion	0.969(0.901-1.010)	0.219	0.991(0.951-1.033)	0.685
Other location	0.952(0.909-0.967)	***0.036***	0.962(0.925-1.000)	0.051
Histology				
Nonfunctional	Ref			
Functional	1.015(0.976-1.055)	0.457		

CI, confidence intervals. The bold values mean the difference between the groups was statistically significant.

## 4 Discussion

Clinical evidence regarding the optimal timing of surgery is lacking for small pancreatic neuroendocrine tumors (usually defined as tumors with a diameter of less than or equal to 2 cm), and some of these tumors lead to progressive disease. Therefore, although tumor size is often used to predict the likelihood of invasion and distant metastasis and, thereby, patient prognosis, the risks associated with the presence of pNETs with a diameter less than or equal to 2 cm need to be re-evaluated.

In this study, 1028 people (31.52% of the total number) were treated without surgery, and the SEER registry recorded patient acceptance of treatments, including local tumor resection, partial resection of the pancreas, radical or expanding pancreaticoduodenectomy, and pancreatectomy. However, the adoption of observational strategies has been reported to be safe for the management of sporadic small pNETs, particularly in single-center studies. Kurita et al. reported no deaths due to small pNETs in a cohort of 172 patients (14), and another case–control study published in 2016 also confirmed that nonsurgical observational management was also reasonable for patients with small pNETs (the median tumor diameter in the observation group was 1.2 cm), with no progression of most tumors (7). However, the results of multicenter studies have shown the shortcomings of the observation method. For example, Chivukula et al. analyzed approximately 2500 cases and found that among patients with small pNETs (15), the five-year survival rate of those who underwent surgery was significantly higher than that of those who did not undergo surgery. In this study, surgery was shown to improve the survival of patients with pNETs that were 2 cm or less in diameter. Regardless of tumor stage, patients who underwent surgery had better OS and CSS than those who did not undergo surgery.

The benefit of surgical treatment may be due to the elimination of the potential malignant and aggressive behavior of the tumor through resection. In 2017, a systematic review reported that although pNETs that were small were relatively safer than those that were larger than 2 cm in diameter with regard to distant metastasis, vascular invasion, degree of differentiation, and tumor stage, no benefit of a “watch and wait” management strategy was found in meta-analyses of studies performed on these types of tumors (10). Surgeons’ concerns about the resection of pNETs mostly stem from the reduction in the benefit to patients caused by surgical complications. A multicenter retrospective study in Germany confirmed that the incidence of postoperative complications with a Clavien–Dindo grade greater than grade III in patients with small pNETs was 8.4% (7/84). The incidence of a grade C pancreatic fistula was 8/84 (9.5%). There were no significant differences in the risks of postoperative complications and the occurrence of postoperative pancreatic fistulas in patients of different ages and sexes, those with different ASA grades and those treated with different surgical methods (16). In addition, previous studies suggested that advanced age increases the risk of complications of pancreatic surgery; consequently, the proportion of patients undergoing active surgical treatment for some diseases is smaller in the elderly subgroup than in the young subgroup (17). Stefano Partelli et al. reviewed the records of patients with pNETs smaller than 2 cm in diameter and found that a younger age was a motivating factor for the selection of surgery as the treatment strategy (18). In this study, the proportion of elderly patients in the nonsurgical group was higher than that in the surgical group. However, regardless of age, surgery can benefit patients in terms of OS and CSS, which indicates that some surgical treatments are needed to prolong patient survival.

The biological behavior of pNETs is less malignant than that of pancreatic ductal adenocarcinomas. Therefore, some studies suggested that aggressive surgery should be performed even for resectable or locally progressive pNETs, and even R1 resection can improve survival (19). Surgical management of metastatic pNETs remains controversial; although resection of the primary tumor and metastases should be considered for metastatic pNETs that can be completely resected according to NCCN guidelines (20), specific benefits for patients with M1 pNETs have not been reported. Some studies have pointed out that patients with gastroenteropancreatic metastatic NETs can obtain survival benefits from surgery that removes at least 70% of the tumor (21). Chakedis J et al. reported the overall survival of 581 patients with M1 gastroenteropancreatic NETs, and the survival duration of patients who underwent surgical resection was longer than that of patients who underwent nonsurgical treatment (22). These results are consistent with those of the analysis of the survival of patients with M1 tumors who underwent surgery in this study, which confirmed that patients with M1 pNETs benefit from surgery.

Since the characteristics of tumors are closely related to patient prognosis, we analyzed the relationship between clinical characteristics and patient prognosis in this cohort of patients. Due to the different developmental processes of the pancreatic head and body/tail (23), the clinical features and prognosis of tumors(pancreatic cancer and pNETs) located in the head and the body/tail of the pancreas are different. In this study, among all pNETs with a diameter less than 2 cm, those in the pancreatic head were associated with the worst prognosis, which was consistent with our previous findings (24). Because local tumor invasion is closely related to tumor size, as reflected in the 2016 ENETS guidelines for the management of pNETs that are less than 2 cm in diameter and located in the pancreatic head, the trend toward the conservative strategy of observation has increased in clinical practice, with pancreaticoduodenectomy being used only in some cases (25).

It is well known that poorly differentiated tumors have more aggressive biological behavior. Previous reports have found stated that some T1 pNETs are well-differentiated, and a temporary delay in surgery does not lead to a worse prognosis. This study also confirmed that better differentiated tumors are associated with a better prognosis. Poorly differentiated or undifferentiated tumors are clear risk factors for lymph node metastasis, local invasion and distant metastasis, leading to a significantly worse patient prognosis (14). A multicenter study in Korea also found that the presence of G2 tumors was an independent risk factor for the metastasis and recurrence of small pNETs, when compared with the presence of G1 tumors (26). A preoperative evaluation of the degree of tumor differentiation is helpful to determine the optimal treatment strategy. Intratumor calcification and lymph node metastasis detected by CT or CT-texture analysis (CTTA) are closely related to the degree of tumor differentiation (27).The fractal dimension of enhanced CT imaging has high sensitivity for the prediction of stage G2/G3 pNETs (28). In addition, endoscopic ultrasound-guided fine needle aspiration biopsy can provide more accurate pathology for pNETs (29).

In this study, approximately 24% of the tumors developed lymph node metastasis, regional organ invasion or distant metastasis. Since some patients did not undergo surgery and pathology could not be obtained to determine whether there was invasion into adjacent organs, the real proportion of patients with invasive tumors may have been higher. Previous studies also showed that lymph node metastasis is associated with worse recurrence-free survival at 5 years (30). Other studies have confirmed that tumor size is closely related to invasion and metastasis. Kwon et al. reported that among 918 patients, those with tumors smaller than 2 cm in diameter had a lower WHO classification and less metastasis, although the clinicopathological features of tumors less than 1 cm and those of tumors 1–2 cm in diameter were similar. However, the rate of lymph node metastasis was higher when the tumor diameter was 1 to 2 cm, whereas no lymph node metastasis was seen in patients with tumors less than 1 cm in diameter (31). In this study, univariate regression confirmed that small pNETs larger than 1 cm in diameter were relatively more invasive, with increased risk of regional invasion and distant metastasis; however, a tumor diameter larger than 1 cm was not a risk factor for invasion, according to multivariate regression. In combination with our previous studies on larger (> 2 cm) pNETs, we found that factors such as the degree of differentiation and anatomical location were more strongly associated with the aggressiveness of small pNETs, which indicates that grouping tumors according to size (</= 2 cm) may not lead to the identification of differences in characteristics in multivariate regression. It has been reported that the characteristics of metastasis are different between pancreatic tumors originating from the ventral versus the dorsal pancreas (32). Small pNETs in the body/tail of the pancreas are less biologically aggressive than those in the head of the pancreas in terms of lymphatic metastasis and local invasion, which may be related to the relative simplicity of the anatomical site and the absence of closely adjacent tissues and organs. It has also been reported that lymph node metastasis may be related to the proximity of tumors to major blood vessels (33). This study also found that the risks of lymph node metastasis and local invasion were lower in patients with small pNETs in the pancreatic body and tail, but the risk of distant metastasis was relatively lower in patients with M1 tumors in the pancreatic body. Functional pNETs have been reported to be associated with a slightly higher risk of lymph node metastasis than nonfunctional pNETs (34), and the NCCN guidelines have recommended lymph node dissection regardless of tumor size in patients with functional pNETs other than insulinomas (20). Therefore, functional tumors are associated with a relatively higher risk of lymph node invasion. This study also confirmed that the presence of functional tumors is a risk factor for lymph node and regional invasion, thus highlighting the importance of the risk of metastatic even in patients with small functional pNETs.

This is one of the largest studies to examine the clinical characteristics of and risk of invasion associated with pNETs that are 2 cm or less in diameter. The aim of this study was to identify a series of factors that can help clinicians make better decisions regarding patient treatment. A few previous articles based on SEER registry data have investigated the benefit of surgical treatment for pNETs staging cT1N0M0 (35). However, this paper focuses on the aggressiveness of small PNETs and analyzes the larger scale data. At present, a prospective, non-randomized, international, multicentre, cohort study on non-functional pNETs not larger than 2cm is being carried out. Part of the results of the mid-term report showed similar characteristics to the present study, such as a lower proportion of elderly patients undergoing surgery, and the presence of 4 patients with M1 in the surgical treatment group also confirmed the potential of distant metastasis of this type of tumor (18). The characteristics of pNETs mentioned in this study may indicate some potential small aggressive tumors, but if necessary, a needle biopsy to determine the nature of the tumor is still warranted. The endoscopic ultrasound-guided fine-needle aspiration biopsy (EUS-FNA) and endoscopic ultrasound contrast-enhanced fine-needle aspiration (CH-EUS-FNA) can effectively determine the degree of differentiation of tumor tissue for grading and risk assessment. When the tumor was larger than 1.5cm, CH-EUS-FNA showed better diagnostic sensitivity than EUS-FNA (36, 37).

As this was a retrospective study based on samples taken from a database, this study has certain limitations. The tumors could not be classified into G1-G3 stages because the SEER database did not record detailed pathological information. The expression of Ki-67, CgA and other markers that affect the prognosis of pNETs could not be obtained from the SEER registry. Such data limited the characterization of tumor in this manuscript. Further pathological data is needed to supplement the results of this study. Moreover, the specific surgical methods used for the partial pancreatectomies were not described in detail, and surgery-related complications were not recorded. Therefore, the relationship between the details of the surgical strategy and the prognosis of patients was not analyzed in this study.

The patients with pNETs ≤ 2 cm in diameter in surgical group have better survival. Age, tumor location, and pathological grade were associated with survival. Small pNETs still could be aggressive, and invasive behavior leads to worse OS and CSS. Risk factors for tumor invasion need to be considered to facilitate clinical decision-making regarding treatment.

## Data availability statement

The original contributions presented in the study are included in the article/supplementary material. Further inquiries can be directed to the corresponding authors.

## Ethics statement

Ethical review and approval was not required for the study on human participants in accordance with the local legislation and institutional requirements. Written informed consent to participate in this study was provided by the participants’ legal guardian/next of kin.

## Author contributions

WM and FC designed the study. WM and JDL acquired the data. CQ and ZF analyzed and interpreted the data. WM wrote the paper. JL and FL critically revised the manuscript for important intellectual content. JL and FL provided financial support. All authors contributed to the article and approved the submitted version.
